# Strengthening regulatory capacity for gene drives in Africa: leveraging NEPAD’s experience in establishing regulatory systems for medicines and GM crops in Africa

**DOI:** 10.1186/s12919-018-0108-y

**Published:** 2018-07-19

**Authors:** Barbara Glover, Olalekan Akinbo, Moussa Savadogo, Samuel Timpo, Godwin Lemgo, Woldeyesus Sinebo, Sunday Akile, Silas Obukosia, Jeremy Ouedraogo, Margareth Ndomondo-Sigonda, Muffy Koch, Diran Makinde, Aggrey Ambali

**Affiliations:** 1 NEPAD Agency, Industrialization, Science, Technology and Innovation Hub, Midrand, South Africa; 2 African Biosafety Network of Expertise, Ouagadougou, Burkina Faso; 3 African Biosafety Network of Expertise, Dakar, Senegal; 4 African Biosafety Network of Expertise, Kampala, Uganda; 5 Simpot Plant Sciences, Boise, ID USA

## Abstract

The New Partnership for Africa’s Development (NEPAD) Agency recognizes that Africa is in a period of transition and that this demands exploring and harnessing safe advances made in science-based innovations including modern biotechnology. To advance the science of biotechnology in Africa effectively, while at the same time safeguarding human health and the environment, the African Union (AU) adopted a High-Level Panel report on modern biotechnology entitled, *Freedom to Innovate*, which advocated for a coevolutionary approach where technology development goes hand in hand with regulation. Furthermore, most AU member states are Parties to the Cartagena Protocol on Biosafety (CPB), a legally binding international agreement negotiated, concluded and adopted within the framework of the Convention on Biological Diversity. This seeks to guide Parties in developing systems for the environmentally sound management of modern biotechnology applications. Currently, 49 AU Member States have signed and ratified the CPB, of which 12 have passed biosafety laws.

African Union (AU) member states are at different stages in the development of regulatory frameworks for applications of modern biotechnology, which include genetically modified (GM) products and other emerging technologies. Biosafety regulatory frameworks comprise: biotechnology and/or biosafety policy; laws, regulations and guidelines; administrative systems; decision-making systems; and mechanisms for public engagement. To assist Member States to implement functional regulatory frameworks for both agriculture and health applications, the NEPAD Agency established the African Biosafety Network of Expertise (ABNE) and the African Medicines Regulatory Harmonization (AMRH).

Currently, transgenic insects and GM crops are regulated by Competent National Authorities whose mandate derives from national biosafety laws. For GM crops, a lot of research has been conducted up to the confined field trial (CFT) and multi-location trials stages in a number of African countries. Burkina Faso has fully functional containment facilities for transgenic mosquitoes while Mali and Uganda are developing theirs. The Burkina Faso regulatory agency has granted permits and has already received sets of sterile mosquito eggs for trials in the contained facility. It is instructive to note that both ABNE and AMRH have worked with national and regional regulatory bodies in Africa to enhance their technical capacities for informed decision making, adoption of best practices, and compliance with international standards. It is against the backdrop of a rich blend of on-the-ground knowledge, experience, expertise, and insight into the context and political sensitivities of member states that the NEPAD Agency seeks to expand existing support. This would include capacity strengthening in the regulation of emerging technologies, such as the application of gene drives in the development of transgenic mosquito for the control of malaria transmission.

## Background

Sachs observed that the world is divided not by ideology but by technology; and that a small part of the globe, accounting for about 15% of the earth’s population, provided nearly all of the world’s technology innovations [1]. A second part, involving about half of the world’s population, was able to adopt these technologies in production and consumption. The remaining part, about a third of the world’s population, was technologically disconnected, neither innovating domestically nor adopting foreign technologies. African leaders sought to change this with the creation of the New Partnership for Africa’s Development (NEPAD) Planning and Coordinating Agency (NPCA), a flagship program of the African Union (AU). NPCA is the technical arm of the AU and seeks among other objectives, to eradicate poverty, place African countries on a path of sustainable growth and development, and to enhance Africa’s effectual participation in the thriving global bio-economy. To achieve these objectives, NPCA established two very important and somewhat related programs. These are the African Medicines Regulatory Harmonization (AMRH) and the African Biosafety Network of Expertise (ABNE). These programmes currently are being implemented on the platform of the NPCA’s Industrialisation, Science, Technology and Innovation Hub (NSTIH) which assists African countries to create enabling regulatory environments that allow science, technology and innovation to thrive especially for agricultural and health applications.

In Africa, political leadership has called for implementation of policies that would enable harnessing science, technology, and innovation safely for economic development**.** At the 27th Ordinary Session of the African Union Summit of Heads of State and Government in July 2016, (https://au.int/en/summit/27) the Assembly endorsed the adoption of emerging technologies for economic development and environmental sustainability. AMRH and ABNE were established in order to create an environment in AU member states where science, technology and innovation can be harnessed to promote Africa’s development.

According to the UNESCO Science report 2010, sustainable development in Africa depends on its capacity to innovate and produce creative technological solutions to address challenges on the continent ([2] b). This is important because Africa continues to trail the world in innovation and scientific outputs [3]. However, there appears to be an increasing appreciation of research and development (R&D) as an engine for development based on the re-commitments by the political leadership to provide resources for agricultural development (10% GDP) and to Science, Technology and Innovation (1% of GDP) [4]. In addition to these policy directives, there is still a need for African leaders to commit to growing the public investment in these areas. Two essential elements for using R&D as a driver of development in Africa are health innovation networks and efficient, sustainable, dynamic linkages and partnerships**.**

Health Innovation Networks help developing countries address neglected diseases. Examples include the Developing Countries’ Vaccine Manufacturers Network and the WHO Developing Countries’ Vaccine Regulatory Network. For technological and social innovation, developing countries need R&D partnerships and implementation of research networks to play a more prominent role in global health [5]. Developing countries should concentrate on areas of comparative and competitive advantage for positive growth, for example, traditional health systems, and areas that will yield the greatest returns.

### Biotechnology applications in Africa

Most of the genetically modified (GM) crops currently commercialised in countries throughout the world have agronomic characteristics (traits) that address biotic stresses, such as pests, weeds, or both with recent growth in traits that address processing and consumer issues [6]. The timeframe for commercialisation of a new GM crop depends on scientific progress in research and development (R&D), field-testing results, and on functioning regulatory systems with science-based decision-making. Regulatory constraints, including non-science based environmental and health impact assessments, have increased in the past decades, delaying approvals and increasing costs [7]. This has sometimes served as a disincentive for investment and use of biotechnology in developing countries by public research organisations, which would rather opt for other less stringently regulated technologies. Thus, the creation of biosafety regulations systems that have clear safety standards and decision-making procedures, and operate in a cost- and time-efficient manner, would support R&D development.

The next generation of precision gene editing tools has developed crops that are moving through field trials into commercialisation. These tools include clustered regularly interspaced short palindromic repeats (CRISPR-Cas9) [8–10], oligonucleotide-directed mutagenesis (ODM) [11], transcription activator-like effector nucleases (TALENS) and zinc-finger nucleases (ZFN) [12–14]. The gene editing mechanisms offer possibilities to increase performance in various sectors including health, agriculture and environment. In particular, they have facilitated the development of gene drive mechanisms, that stimulate biased inheritance of a particular gene to alter populations at the release site and these are being proposed for changing local populations of harmful organisms such as mosquitoes [15–17].

As suggested in previous articles [18], gene drives could be applied to prevent mosquito populations from transmitting the malaria parasite or to suppress a local mosquito population. This is of immediate interest to Sub-Saharan Africa where malaria still represents the most harmful threat to public health. Optimistic estimates predict that releasing mosquitoes with these traits at just 1% of the total wild population of mosquitoes that vector malaria could eradicate malaria within a year [18, 19]. Sub-Saharan Africa region is the most affected by malaria parasites that are transmitted by the *Anopheles gambiae* complex mosquitoes. Despite all efforts deployed over the number of decades, millions of people, including young children, are still dying of malaria or at best remain so badly affected that they only survive with reduced capability to contribute to the economic growth in their respective countries. However, significant progress has been made over the last decade that led to a drop of over 50% in the number of cases in more than 40 African countries since 2001. Vector control, mainly with the use of Long Lasting Insecticide Nets (LLIN) and Indoor Residual Spraying (IRS) has played a major role in this [20]. Unfortunately, however, residual levels of transmission persist even in places where the coverage rate of these intervention tools is above 80%. The failure of these vector control measures to completely eliminate transmission may be due to several factors, including insecticide resistance. Expert opinion suggests that unless additional tools/strategies are found to deal with residual transmission, malaria elimination will be out of reach no matter how much effort and resources are deployed [20].

### Changing regulatory landscape for biosafety in Africa – Role of ABNE

#### Core guiding principle and key strategic areas of intervention

The NEPAD Agency established the African Biosafety Network of Expertise (ABNE) in 2008 in partnership with Michigan State University, USA, and with funding from the Bill and Melinda Gates Foundation. ABNE’s mandate is to assist building functional regulatory systems in AU member states for the safe and responsible application of agricultural biotechnology [21].

**Fig. 1:**
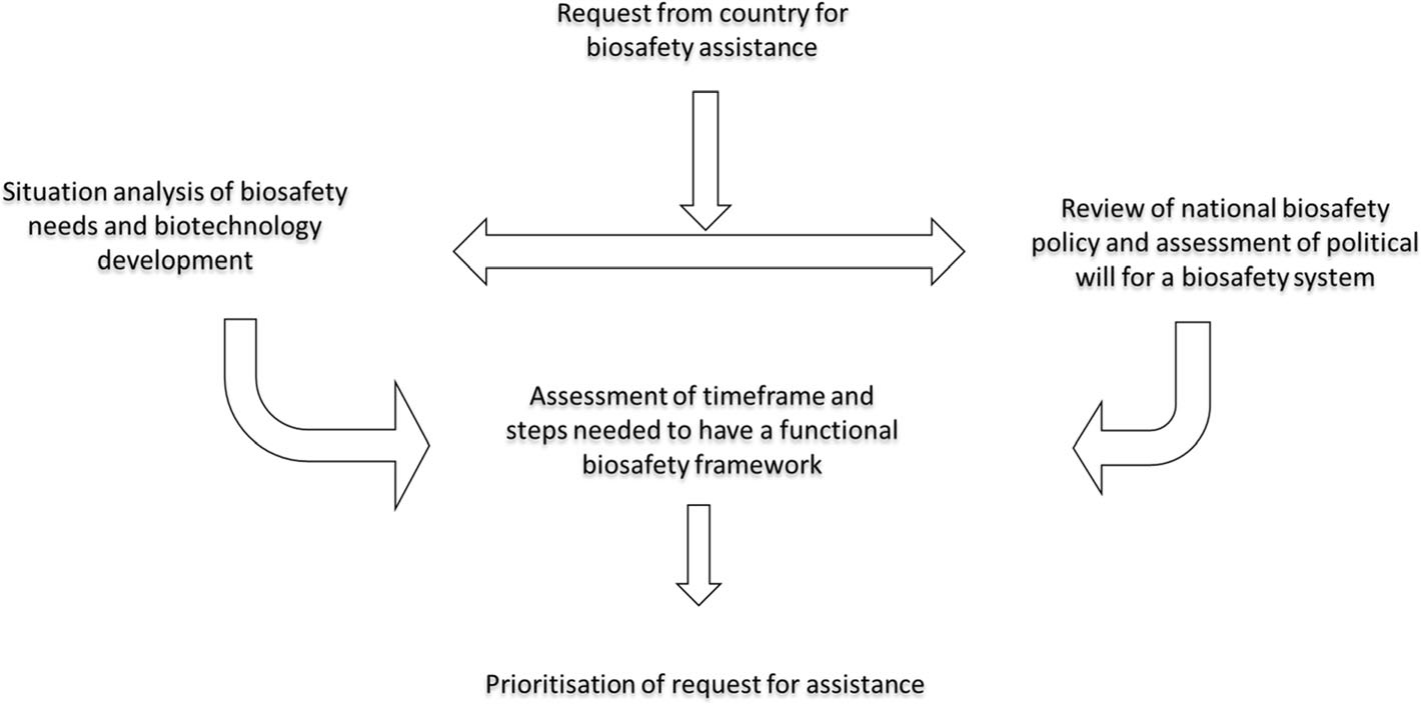
Initial steps in a roadmap for effective biosafety intervention

Since 2010 ABNE has worked with African countries to develop and implement functional national biosafety frameworks. This experience has provided insight into effective ways to assist countries and these lessons are presented here in the format of a roadmap for effective biosafety intervention (Fig. 1). ABNE key areas of intervention include: assisting member states in developing policies and regulations to promote safe development, diffusion, and adoption of agricultural GMOs; conducting training and workshops on risk assessment techniques and their application to inform decision-making; training to improve critical mass of regulators with enhanced competencies in biosafety regulation; enhanced biosafety communication and cooperation among member states; and exposing regulators to best practices under the south-south/ north-south cooperation.

#### Lessons learnt from establishing biosafety systems in Africa

##### Prioritization is essential

At the start of the ABNE project, a needs analysis was carried out with 18 African national biosafety focal points. Survey countries were selected based on a set of criteria that included the presence of active agricultural biotechnology development and/or deployment programmes that would need a functioning biosafety system in the near future; the presence of institutional requirements for a national biosafety framework, and political will to implement a national biosafety system.

Results from the survey informed selection of a few countries as focus countries that would receive specific intervention support. In addition, generic biosafety services were offered to the other member states. The focus countries were identified as being the most likely to require biosafety decision making in the immediate future and which had the political will to implement the framework needed to enable these decisions.

One of the first considerations in evaluating requests for assistance was a review of the existing policy in the country and whether the capacity building was for biosafety activities that could be implemented in the country (Fig. 1). In many instances, policy gaps were identified and it was necessary to work in parallel on policy revision and additions. The first activities requested by partner countries were not always the logical starting points for building biosafety capacity. Working with country focal points to review requests and identify where to start, required expertise and diplomacy. Prior to the implementation of in-country and regional intervention programmes, NEPAD Agency ABNE Programme Officers with the assistance of regulators and policy makers within the national systems, conducted on-the-ground assessments in the focus countries to establish priority needs and develop a joint programme of work to achieve clearly spelled out goals.

##### Biosafety policies must be workable

Countries that are successful adopters of agricultural biotechnology have sound national biosafety policies and supportive biosafety systems. Good examples are South Africa, Ghana and Nigeria. The first priority is the development of national biosafety policy that supports national institutions to provide transparent biosafety decision-making. Biosafety policies in some AU member states do not reflect global experience as they focus on risk and do not implement basic risk assessment and risk management to enable safe access to benefits. Other systems include non-safety considerations such as socioeconomic considerations in risk assessment without sufficient data, or have strict liability clauses, which deter not only the technology developers, but also the public research scientists wanting to experiment with GMOs. Often, in non-functional regulatory systems, the cost of regulatory requirements are unaffordable or even unenforceable, and there are potential conflicts that arise between national biosafety laws and evolving regional harmonized regulatory policy. For instance, in the COMESA region where a biotechnology policy has been developed and adopted, regulatory Acts in some member states resulted in the policy becoming unimplementable.

It is not necessary to have national biosafety legislation in place before initiating biotech research and development (R&D). Countries have used interim legal instruments to enable R&D and field-testing of GM crops, as in Ghana. Contained and confined R&D can be carried out safely under existing provisions for science, technology and innovation, including quarantine requirements under departments of agriculture. A national biosafety authority can work within these provisions to establish policy and guidelines for GM R&D. Uganda is the leading country in Africa conducting CFT on many indigenous commodities and using policy and guidelines for the approval of CFT. Nigeria started CFTs well before the Biosafety Act was gazetted in 2015. Under these provisions, national biosafety authorities in Africa have approved GM R&D facilities and received, reviewed, and made decisions on applications to run CFT with GM plants and microbes. This experience and expertise helped develop workable legal instruments for the general use of GM crops and food and feed imports of GM products.

In most countries, the legal provisions for general release of GM crops and for GM imports were developed and passed while GM R&D continued. Importantly, it takes many years to pass laws and where existing laws could be used for the development of regulations specific to GM testing and release, these were used, or adapted and used.

In many countries, existing legal instruments have enabled an interim biosafety process to run, providing biosafety officers with experience in handling applications, issuing permits, inspecting for compliance and developing workable processes and guidelines for developers and users of agricultural biotechnology and GM crops. The importance of transferring this experience in the transition to any new biosafety institutions and bodies must be emphasised. In countries where there was not a deliberate effort to transfer existing expertise and experience in new biosafety structures (e.g. South Africa and Kenya), there were considerable delays in getting the new institutions functional and efficient. In countries where the institutional capacity was transferred to new biosafety bodies (e.g. Ghana’s National Biosafety Authority and Nigeria’s National Biosafety Management Agency), the institutional history and experience helped to ensure that the biosafety functions were not impacted by indecision, misinterpretation of provisions, and administrative delays. The transition to new biosafety institutions is considered positive progress when the new institutions are up and running effectively and quickly. In countries where misinformation had left biosafety officers with negative and incorrect assumptions about the safety and value of GM crops, the transition to new structures gave the government the opportunity to staff new institutions with informed scientists confident in their ability to apply risk assessment and risk management for the benefit and safety of local people and environment.

Passing a new biosafety law has proved relatively simple and quick in countries where the need for the law is understood by the lawmakers (e.g., Burkina Faso). However, for most other countries, the process is long and convoluted. Where lawmakers are not informed about the need for the legislation or are misinformed through the actions of activists, the protracted process takes five or more years, even with a steady push from interested and affected science stakeholders. Moving these processes forward is a huge challenge for many countries.

Experience suggests that science is not the best motivator for passing a biosafety law. Instead, the motivation needs to come from the stakeholders who want access to improved GM technology. For GM crops, this is the farming sector. Unless farmers’ organizations express a clear need for access to improved planting material, there is no real push for the new biosafety laws. Similarly, unless the national environmental sector wants the country to benefit from the environmental sustainability of GM crops, there is no push for new biosafety legislation. Finally, unless consumers want access to safe and affordable food derived from GM crops and technology, there will be no push to pass biosafety legislation. Clearly, science and scientists alone cannot motivate effectively for biosafety legislation.

During the period where legal instruments are reviewed, finalised and passed, it is valuable to initiate biosafety capacity building for all aspects of biosafety oversight. Implementing procedures for contained and confined R&D builds experience with risk assessment, risk management and risk communication that is valuable to developing workable legislation for general use of GM organisms. While there is some concern that many of those trained in the interim years are no longer employed within the system once the formal biosafety process is implemented, it is important for legislators to know that the country has the capacity and experience to implement new legislation. National biosafety offices are encouraged to document trained personnel and ensure that they are given an opportunity to use their biosafety skills after training activities.

Different jurisdictions have adopted legal regimes that best suit national legislation. Some countries have enacted specific laws to regulate the safe use of biotechnology. Other countries have used parts of existing legislation for R&D, agriculture, quarantine, food safety, feed safety, etc., to provide inter-ministerial jurisdiction for regulating GMOs.

Legislative requirements include: objectives, explanatory preambles for policy context, definitions, administrative structures, risk assessment and risk management for biosafety activities, decision-making, regulatory compliance, enforcement powers, liability and redress, appeal structures, socioeconomic considerations, and mechanisms for public participation. Biosafety administrative systems receive and handle applications for contained, confined and general release of GMOs, including export, import, and transit.

Some challenges in national biosafety regulations include:

- Unclear objectives and scope. Mixing environmental safety and food safety has required unnecessary Environmental Risk Assessment (ERA) for import of non-living products derived from GMOs;
- Inconsistent definitions. Definitions need to be consistent with those in CPB, *Codex Alimentarius,* and existing laws;
- Unworkable and impractical provisions. For example, not requiring science-based risk assessment and decision making has delayed decisions; and not combining CFT approvals with import permits for the research materials has complicated administrative tasks for research;
- Lack of regulatory transparency***.*** Absence of required information on the Biosafety Clearing House makes it difficult to navigate and predict the regulatory process, including determinations of which laws take precedence when GMOs fall into several regulatory jurisdictions;
- Disproportionate clauses***.*** For example requiring ERA for short term CFTs when these data are not available or relevant for this level of release. Or, having strict liability for any kind of damage which may warrant prison sentences for administrative errors; and
- Funding***.*** The most common constraint is the limited operational budget in most biosafety focal points needed to carry out their responsibilities, such as performing effective evaluations and review of dossiers, to arrive at independent defensible decisions.

##### Timely biosafety training

Once legal changes are approved by stakeholders and the amended provisions are approved through the legislative procedures, biosafety training should start. This will ensure that capacity exists in the national biosafety structures to process applications for field trials, food imports and general release.

In the past, biosafety capacity building has been used as a platform to review biosafety legislation and create awareness needed to pass legislation. However, this may not be the most effective way for moving biosafety legislation through law making procedures. As noted above, farmers, environmentalists and consumers are the best motivators for new legislation. Obtaining a balance between training too early and effective motivation for lawmakers should be reassessed because, while having technical competence during the drafting of legislation and regulations is important, this may be achieved without extensive national biosafety capacity building. However, the capacity building trainings designed in the past focus on the key stakeholders such as regulators such as members of the national biosafety committee (NBC), institutional biosafety committee (IBC) and quarantine officers, scientists, study tours for decision-makers.

There is a narrow line to walk when working with national legislation. How does the NEPAD Agency support this process without being seen to be interfering? To achieve this the NEPAD Agency strives to establish a strategic position that remains neutral to the outcome, bearing in mind that if legislation remains restrictive there will be no need for biosafety infrastructure and processes and, therefore, no need for biosafety capacity building in the country. NEPAD Agency strives to maintain their credibility through evidence-based facts with national authorities. ABNE provides opportunities for key government gatekeepers to visit other countries and see best practices in terms of GM crops and what is meant by functional regulatory systems. These study tours are referred to as “seeing is believing” tours.

##### Amending/ reviewing biosafety laws

From experience we know that amending laws is often a long process that requires two key steps: ensuring that the technical wording of the policy is accurate, implementable and consistent with other national laws; and moving the amended policy through established, mandated administrative and legal processes.

Both steps are required, but for the process to be completed in a timely manner, depending on the country dynamics, actors have had varying degrees of success. The NEPAD Agency ABNE has been mostly successful at the first step, but less effective at helping local biosafety stakeholders move amended legislation or policy through government approval processes, as these are entirely national processes that must not be influenced by undue external pressure.

The NEPAD platform has been effective in garnering regional political support for biosafety processes and policy requirements. African countries have accepted the Recommendations in the publication *Freedom to Innovate Biotechnology for Africa: Report of the High-Level African Panel on Modern Biotechnology* that acknowledges the value of biotechnology in economic development in Africa, and they have committed to spend more on science, technology and innovation, so support for biosafety policy gives them a mechanism to act on these convictions and promises [22]. Proceeding diplomatically requires an approach that says, ‘*how can we help’*, rather than *‘do this’*.

Biosafety regulations should be tools for informed decision making that allow countries to take advantage of all biotechnology techniques to assist with national and local challenges. In short, biosafety regulations are not an end. They provide a precautionary process that maximizes access to the benefits of modern biotechnology by minimizing potential risks through scientific risk assessments. Approvals are given with or without conditions to manage risk (i.e. risk management measures).

##### AMRH platform as a model for regulating gene drives in Africa

The African Medicines Regulatory Harmonization (AMRH) Programme was initiated as a strategy to mitigate capacity limitation challenges faced by the majority of national medicines regulatory agencies (NMRAs) in Africa to carry out basic regulatory functions. A wide range of factors contributed to limited regulatory capacity; from lack of or outdated legislation, regulations and guidelines that meet internationally acceptable standards, to limitations in terms of human and financial capacity and lack of the necessary infrastructure to execute their mandate. In recognition of these limitations and taking into account the important role NMRAs play in providing an enabling environment for investment in the pharmaceutical sector, the African Medicines Regulatory Harmonization (AMRH) Programme was initiated in 2009, as part of the implementation of the African Union Pharmaceutical Manufacturing Plan for Africa within the NEPAD Framework [23]. With the support of our development partners, tremendous progress has been attained to date. While the partnership aimed at galvanising the needed political, technical and financial support, regional economic communities (RECs) and regional health organizations (RHO) are benefiting from harmonized regulatory requirements, standards and practice. These facilitate faster approval of quality, safe and efficacious medical products and technologies and at the same time assuring expanded regional markets for the industry.

With the launch of the East African Community (EAC) medicines registration harmonization project (MRH Project) in 2012, the Economic Community of West African States (ECOWAS) region through collaboration between the West Africa Health Organization (WAHO) and the West African Economic and Monetary Union (WAEMU); and the Southern African Development Community (SADC) have also embarked on implementation since 2015. Countries are now conducting a joint assessment of products with subsequent approval at the national level in a much faster way. From the initial focus on the harmonization of requirements for marketing authorization of products, the programme is been expanded to cover safety surveillance and control over clinical trials. These works are done through established governance structures involving regional Expert Working Groups and Steering Committee composed of members from the NMRAs.

Other equally important developments include the endorsement by the AU Summit in January 2016 of the Model Law on Medical Products Regulation, which has been utilised by six (6) countries [24]. They include Lesotho, Zimbabwe, Ivory Coast, United Republic of Tanzania (Zanzibar), Seychelles and the Gambia. The Model Law serves as a comprehensive guide to member states in the review and/or development of national legislation and as a framework to support member states and regional economic communities (RECs) in their endeavour to harmonise medical products regulation. It further provides an enabling regulatory environment for the private sector to deliver quality, safe and efficacious medical products and technologies to the African population.

The designation of eleven Regional Centres of Regulatory Excellence (RCOREs) since 2014 to provide academic and technical training in regulatory science in various regulatory specialities is also another undertaking of the AMRH Programme [25]. The overall goal of RCOREs is to increase the regulatory workforce in Africa through partnerships between NMRAs and academic and/or research institutions; with specific regulatory science expertise as well as training capabilities. RCOREs have played a critical role providing training programme on various regulatory areas, through hands-on training, twinning and exchange programmes among NMRAs. There has been progress in streamlining training curricula on Clinical Trials Oversight. For instance, a draft manual on Clinical Trials Regulatory Fellowship Training, under the leadership of the Ghana Food and Drug Authority, University of Ghana’s School of Public Health, and in collaboration with other RCOREs, has been developed.

Cross learning between biosafety and medical products, regulatory systems strengthening and harmonization initiatives are critical for developing regulatory capacity in gene drive technology, funding this type of activities in Africa through continental body helps in the oversight and commitments of the member states.

##### Regional efforts to harmonize biosafety frameworks

The Regional Economic Communities (RECs) in Africa group individual countries in sub-regions for the purposes of achieving greater economic integration. They are the ‘building blocks’ of the African Union (AU) and are central to the strategy for implementing the NEPAD Agency programmes. There are nine sub-regional blocks: Arab Maghreb Union (AMU); Common Market for Eastern and Southern Africa (COMESA); Community of Sahel-Saharan States (CEN-SAD); East African Community (EAC); Economic Community of Central African States (ECCAS); Economic Community of West African States (ECOWAS); Intergovernmental Authority on Development (IGAD); Southern African Development Community (SADC); and West Africa Economic and Monetary Union (WAEMU). The role of Regional Economic Communities will bring value to the regulation of borderless technology, like GM mosquitoes, that requires harmonized regional regulatory coordination for safe and effective deployment.

**Fig. 2:**
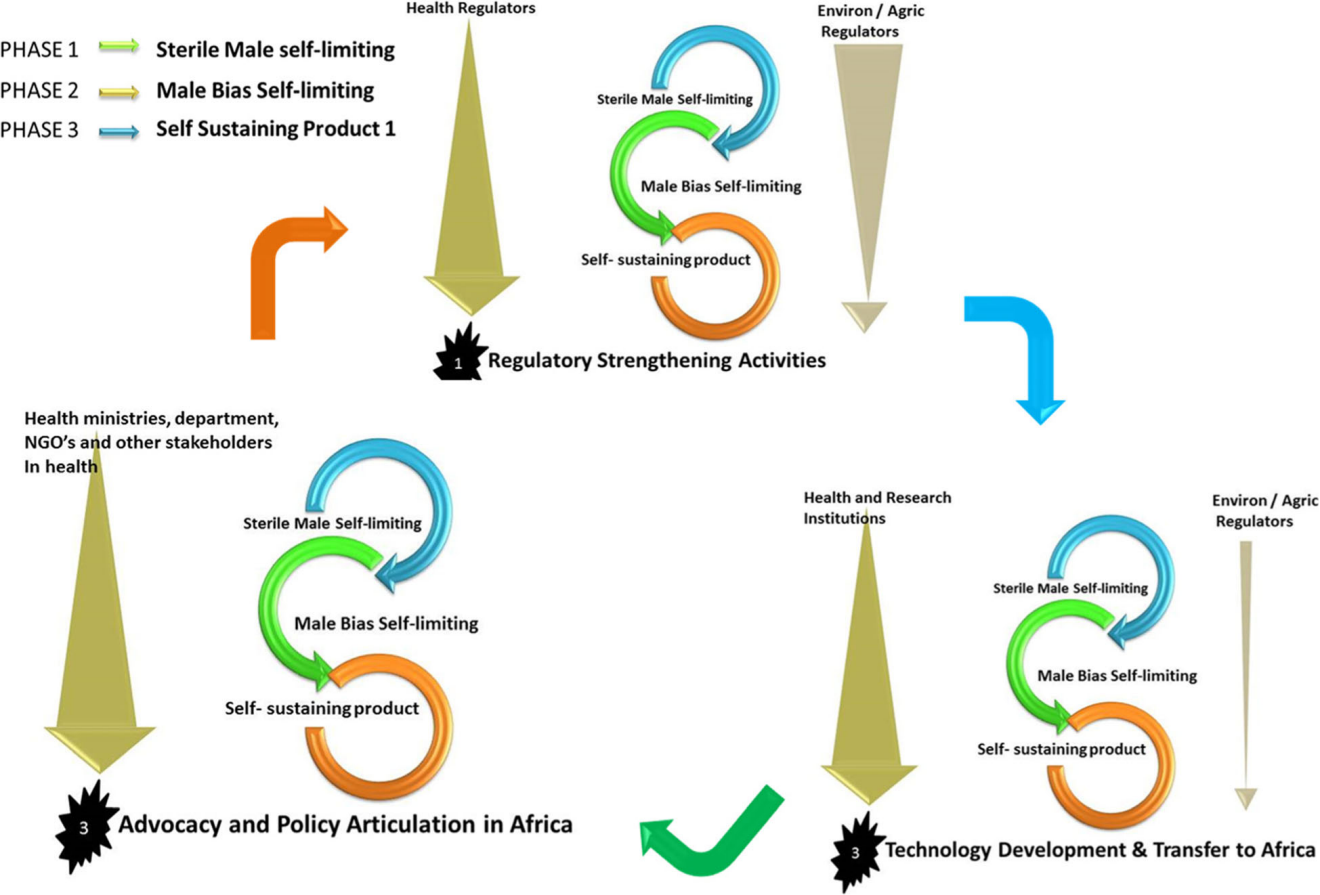
Environmental and health regulator roles in gene drive regulatory strengthening, policy advocacy, and technology development transfer

Through its two specialized institutions, AMRH and ABNE, the NEPAD Agency has obtained experience in facilitating national regulatory capacity building and sub-regional regulatory harmonization for medicines and for agricultural biotechnology [26]. In Fig. 2, the environmental regulator’s role is central in the assessment of the technology from the onset, focusing on risk assessment to demonstrate the safety of the technology in the environment including transboundary movement. Health regulators will assess health benefits in the suppression of malaria vector to humans. NEPAD Agency is:

- Supporting the establishment of relevant regulatory bodies;
- Conducting stakeholder empowerment through “seeing-is-believing” study tours;
- Supported the development and review of policies and guidelines;
- Advocated for and deepened community engagement processes; and
- Supported public education and awareness creation.

The AMRH has developed a regional platform for regulating medical products and technologies working through regional economic communities. There is an existing structure for African regulators at the regional level, especially those in health, which can be used to build trust, confidence, ownership and alignment as countries develop regulatory systems for targeted malaria control.

Releasing living, genetically modified organisms into the environment requires careful risk assessment and risk management to understand and manage the cascade of population dynamics and evolutionary processes that could have environmental impacts. Biosafety regulators are trained to undertake these assessments and will be strengthened by training on existing standard operating procedures (SOPs) applicable to gene drive modified organisms. As for GM crops, the risk decision takes into account potential benefits and harms, including risk management approaches to reduce or mitigate any identified potential harm [27]. NEPAD Agency’s experience in supporting regulatory systems will be useful in regulating mobile, living modified organisms.

NEPAD Agency will combine the experience it has gained in facilitating approaches to regulating pharmaceuticals and GM crops to build the regulatory capacity required to evaluate gene drive application such as might be used to eradicate malaria through control of the disease vector mosquitoes by:

1. Building on prior progress and existing capacity built in part by ABNE programmes.
2. Forging strategic partnerships with national and international organizations and programs involved in technology development and strengthening regulatory systems.
3. Providing demand-driven services to national agencies using existing prioritisation processes
4. Being proactive and mindful of national and regional sensitivities.
5. Being flexible with capacity building efforts to accommodate changing and emerging needs.
6. Using regional platforms to help manage the potential for transboundary movement.

These approaches will support communication strategies, effective public awareness and consultation.

### A coordinated regulatory framework for gene drive

**Fig. 3:**
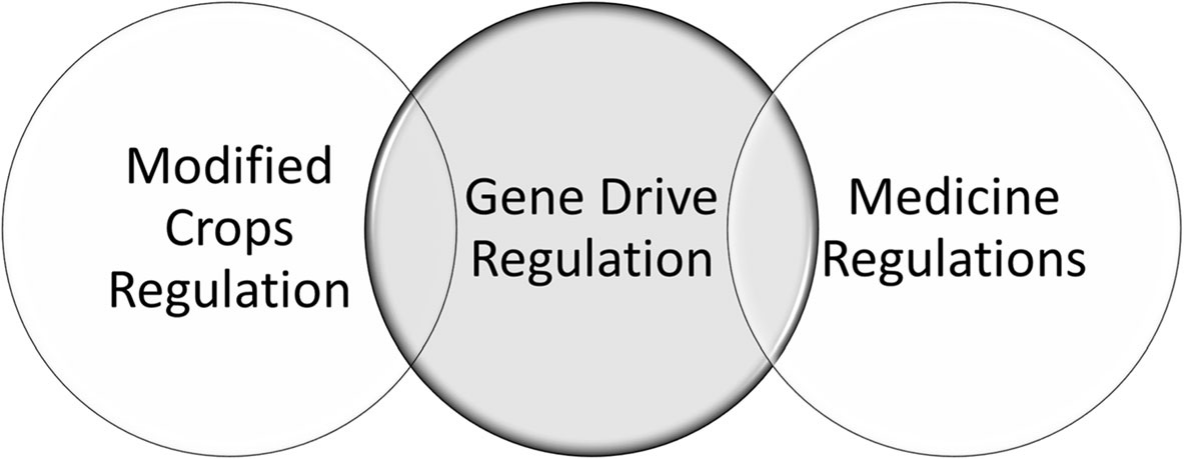
Schematic diagram of a Coordinated Regulatory Framework for Gene Drives

As indicated in the National Academy of Science report on gene drive, gene drive regulation crosscuts institutions and thus requires a coordinated framework that enables various institutions to regulate aspects that fall within their mandate, based on its use [27]. In Fig. 3, transboundary movement of modified crops is regulated under the Article 17 of the Cartagena Protocol on Biosafety. Both the modified crop regulation under the Cartagena Protocol on Biosafety and medicine regulation under the World Health Organization have an overlapping role to play for gene drive regulation.

## Conclusion

The creation of an enabling environment for science, technology and innovation (STI) as well as for research, science, technology and development requires optimising human resources and infrastructure; providing oversight governance for innovation systems; leveraging financial resources; establishing monitoring and assessment mechanisms, and offering advice on STI within African institutions. AU leaders must significantly increase public investments in biosciences R&D. Failure to do so will impair the continents’ capacity to stay connected to global advances in biosciences and to transfer, adapt and explore life sciences knowledge for the benefit of all Africans.

Sustainability of emerging technologies in Africa will require new policy direction, political will, and leadership by government agencies to strengthen biosafety capacity and compliance. As seen in developed countries, low risk of some gene editing techniques may lead governments to implement more effective and timely decision-making processes that are commensurate with low risk.

In the recently conducted consultation meetings held separately across the regional blocs in Africa (ECOWAS, SADC, EAC, COMESA), which participated in problem formulation for gene drive technology; it was overwhelmingly understood that if we had gone the same route for GM technology it would have positively influenced the adoption of the technology. Participants discussed freely expressing their concerns and it was evident at the end of the workshop that the technology will be welcome in Africa. It was emphasized at the workshops that the technology will not be a silver bullet. Therefore, deployment alongside the existing tools used in the fight against malaria is necessary.

The recent advent of new technology has brought hope to crop improvement and disease control. However, there is need to build capacity in many African countries for oversight of the safe adoption of new technologies. Systems and institutions at country, regional and continental levels need training to allow for the optimal use of biotechnology and access to benefits it brings. Many African countries still need to put in place regulations that would enable the safe adoption of biotechnology products. Existing biosafety laws are sufficient for regulating emerging technologies and national implementation can be strengthened by international WHO guidelines on pharmaceutical products.
